# The Influence of Industrial Structure Upgrade on Coupling and Coordinated Development—Empirical Analysis From Chinese Pharmaceutical Manufacturing and Pharmaceutical Service Industries

**DOI:** 10.3389/fpubh.2021.722006

**Published:** 2021-09-14

**Authors:** Meng Hu, Chunhai Tao, Hao Zhou

**Affiliations:** School of Statistics, Jiangxi University of Finance and Economics, Nanchang, China

**Keywords:** medical service industry, pharmaceutical manufacturing industry, coupling coordination degree, industrial structure upgrading, quantile

## Abstract

The degree of coupling and coordination between the pharmaceutical manufacturing industry and the medical service industry crucially requires improvement by promoting the development of the pharmaceutical manufacturing industry through industrial structure upgrading to narrow the gap between them. First, this article uses the coupling coordination degree model to measure the coupling coordination degree of the Chinese pharmaceutical manufacturing industry and medical service industry; then, it theoretically analyzes the mechanism through which upgrading the pharmaceutical manufacturing industry's industrial structure can improve the coupling coordination degree. Finally, we empirically test the impact of upgrading the pharmaceutical manufacturing industry's industrial structure on the coupling and coordination degree between it and the medical service industry. The main conclusions are as follows: (1). The quantile regression model shows that having an advanced pharmaceutical manufacturing industry positively affects its coupling and coordination with the medical service industry; (2). A threshold regression model is tested, and it is found that only when the pharmaceutical manufacturing industry reaches an advanced level can it significantly promote joint and coordinated development with the medical service industry; (3). Rationalizing the pharmaceutical manufacturing industry structure will inhibit a high level of coordination between it and the medical service industry and their coordinated development.

## Introduction

The pharmaceutical manufacturing industry and medical service industry are core components of a country's health industry, and they are also “engines” that promote economic growth. The higher the degree of coupling and coordinated development between the two, the more stable the development of the health industry, and the more competitive the country will be; uncoordinated development between the two will result in economic virtualization and a hollowing out of the health industry, which is not conducive to promoting the sustainable development of public health. The pharmaceutical manufacturing industry and the medical service industry are connected, and only sound coordination between the two as they develop can promote the high-quality development of the health industry. As the most populous country globally, China's huge population base, growing pressure from population aging, and increasing demand for medical services brought about by the improvement of living standards have promoted the rapid development of the Chinese medical service industry. The proportion of government health expenditure in total health expenditure increased from 15.93% in 2004 to 27.74% in 2018. The annual expansion of government health expenditures has also significantly improved the level, quality, and efficiency of the medical service industry, thereby promoting its continuous development. At the same time, with the continuous advancement of policies such as the “two-invoice system,” “consistency evaluations,” and “three-medicine linkage,” China's pharmaceutical manufacturing industry has also developed rapidly, and its output value has continued to expand. However, notably, the pharmaceutical manufacturing industry is developing at a significantly lower rate than the medical service industry. The imbalance in the development speed of the two restricts their combined and coordinated development, leading to a low degree of coordination and annually expanding regional gaps between them (to avoid repetition, hereafter, “the coupling coordination degree” refers to the coupling coordination degree between the pharmaceutical manufacturing industry and the medical service industry). The imbalance in the coupling coordination degree represents an imbalance in the health industry, and this imbalance, in turn, will inhibit regional economic growth (1). The main cause of this restriction on coordinated development is that the pharmaceutical manufacturing industry's development speed lags that of the medical service industry. Therefore, accelerating the pharmaceutical manufacturing industry's development is a meaningful way to improve the coupling and coordination of the two. Under the new economic normal, the rapid development of the pharmaceutical manufacturing industry has begun to experience multiple problems, and its high-speed growth has become unsustainable. Standing at the threshold of reform, industrial structure upgrading is a meaningful way to break through this barrier. Speeding up the industrial structure adjustment of the pharmaceutical manufacturing industry is not only an objective requirement if the Chinese pharmaceutical manufacturing industry is to develop to the next stage but also an inevitable requirement to further promote its high-quality development and its coordinated development with the medical service industry.

Domestic and foreign scholars have conducted in-depth research on upgrading industrial structures from macro and micro perspectives. At the macro level, changes in the proportion of industries (agriculture, manufacturing, and service industries) across society and integration with value chain theory can reflect the overall level of industrial upgrading. From a value chain perspective, exploring the driving force and influencing factors of industrial upgrading can help trade policy promote the upgrading of the industrial structure by improving product quality and enhancing the status of the value chain (2); The “One Belt, One Road” regional value chain can promote development of the domestic value chain and the upgrading of industrial structure (3, 4) combines industrial structure upgrading with value chain upgrading, to explore the impact of industrial value chain upgrades in different countries. Many scholars, such as Fu (5), Deng and Cao (6), Lin and Zhong (7), have researched the industrial structure upgrading path from the perspectives of economic growth, financial structure, and ecological civilization. The meso level mainly explores the upgrading of the manufacturing industry structure. At present, domestic scholars still have different definitions of the transformation and upgrading of the manufacturing industry, and most use the proportion of high-tech industries or total factor productivity to interpret the degree of manufacturing industry upgrading. Song and Zhang (8), Ji (9), Sun (10), Jiangand Wang (11), Fu et al. (12), and Shan (13) explore the driving force of manufacturing upgrading from the perspectives of innovation, technology, the producer service industry, human capital, foreign investment and collaborative innovation. Zhou (14) explores the impact from coupling the manufacturing and service industries on manufacturing upgrades and finds that coordinated development has a non-linear driving effect on manufacturing upgrades. At present, most of the research on manufacturing subindustry upgrading focuses on the equipment manufacturing industry. Wan et al. (15) and Zhang (16) found that intelligence and industrial agglomeration can promote the upgrading of the equipment industry. Despite a focus on microenterprises and production factors, few studies on industrial upgrading have been conducted on the transformation of enterprises from capital- and technology-intensive industries. Cai et al. (17) used the geese model and found that an increase in total factor productivity can promote the upgrading of industries in China. Current research on this topic, such as Zhang et al. (18), Jiang and Xi (19), Francois and Woerz (20), Francois and Hoekma (21), and Li et al. (22), mainly expounds on the industrial coupling and coordination mechanism considering industrial correlation theory, labor division theory, transaction cost theory, and new economic geography theory.

As this review of the existing literature shows, many scholars have conducted research on the impact of the coordinated development of industrial coupling on industrial structure upgrading and have achieved fruitful results. However, there are few studies on the impact of industrial structure upgrading on the coordinated development of industrial coupling, especially in the pharmaceutical industry. Literature on the impact of manufacturing upgrading on collaborative development between the pharmaceutical manufacturing industry and medical service industry is rare (23). However, a certain degree of industrial structure upgrading can effectively promote the development of the manufacturing industry, mainly by advancing and rationalizing the manufacturing industry, which can improve the manufacturing industry's development, promote the coordinated development of industries, and promote an increase in the regional economy (24, 25).

Given the above, this article uses the quantile and threshold regression model to clarify the impact of the industrial structure upgrading of the pharmaceutical manufacturing industry on the degree of coupling and coordination from the two perspectives industry advancement and rationalization. The level of health has important practical significance.

## Theoretical Analysis

According to the “Statistical Classification of the Life Service Industry (2019)” issued by the National Bureau of Statistics, the medical service industry belongs to the life service industry, which mainly includes hospital services, primary medical and health services, and professional public health services. As the primary industry of the medical service industry, the pharmaceutical manufacturing industry provides necessary intermediate products and promotes the overall development of the medical service industry. The medical service industry reduces costs by providing convenient and high-quality medical services. The pharmaceutical manufacturing industry's sales and circulation costs provide favorable conditions for expanding reproduction, optimizing the industrial chain structure, and improving the efficiency of production research and development. The pharmaceutical manufacturing industry and the medical service industry are located upstream and downstream, respectively, in the health industry supply chain. By providing medical products and medical services to meet residents' health needs, they are integrated into the modern service industry and the advanced manufacturing industry. The coupling and development of the pharmaceutical manufacturing industry and the medical service industry experience a cycle within their economic category and will progress from low-level coupling (emergent stage) to low-level coupling (initial stage), medium-level coupling (sound stage) and high-level coupling (Mature stage) Through continuous evolution, the two industries will gradually form a stable dynamic link, extend the health industry value chain, and deepen the high-quality development of the health industry.

The pharmaceutical manufacturing industry's industrial structure refers to its economic components, the composition of its economic activities, and the mutual constraints. According to the “National Economic Industry Classification Standard (2019 Edition),” pharmaceutical manufacturing applies physical or chemical changes to raw materials to create new pharmaceutical products; this includes the manufacture of chemical raw materials, chemical preparations, and Chinese herbal medicine processing. There are seven subsectors of Chinese patent medicine production, including veterinary medicine manufacturing, biological medicine manufacturing, sanitary materials, and medical supplies manufacturing. The upgrading of the industrial structure refers to transforming the industrial structure from a low-level form to a high-level form by adjusting factors such as technology and management. The upgrading of the pharmaceutical manufacturing industrial structure is manifested in two aspects: advancing and rationalizing of the industrial structure. According to the “National Medium and Long-term Science and Technology Development Program 2006–2020,” the development of chemical preparation manufacturing and biomedicine manufacturing is characterized by high addition, high intelligence density, and high-tech content and can thus to some extent represent an advanced level of pharmaceutical manufacturing. According to the theory of resource allocation, at a certain stage of industrial development, through the flow of capital and labor, the industrial structure is continuously adjusted to guide the coordination of factors and associations and realize the rational allocation and effective use of resources between industries: the industrial structure is thus rationalized.

From a micro perspective, product demand promotes coupling and coordination. As the pharmaceutical manufacturing industry's industrial structure gradually advances, the demand for product and technology research and development will further deepen the talent flow between the pharmaceutical manufacturing industry and the medical service industry, create a well-matched interindustry technology chain, and promote the coordinated development of coupling. As the industrial structure of the pharmaceutical manufacturing industry gradually adjusts from unreasonable to reasonable, the demand for high value-added and high-tech products is conducive to encouraging small and medium-sized enterprises to reorganize, merge, and update their technology, thereby forming industrial clusters, promoting synergistic agglomeration between industries, generating a scale effect, reducing the rate of industrial resource loss, optimizing the output of the pharmaceutical manufacturing industry, deepening the integration of the pharmaceutical manufacturing industry with the medical service industry value chain, and ultimately promoting the improvement of coupling coordination.

From a meso-level perspective, deepening the division of labor improves the degree of coupling and coordination. As the industrial structure of the pharmaceutical manufacturing industry gradually advances, enterprise technological innovation leads to the development of advanced products or technologies, which penetrates into the medical service industry, changes the technical route of the pharmaceutical manufacturing industry, deepens its division of labor, optimizes its production costs, and increases its production efficiency. The mutual penetration of the medical service industry and the pharmaceutical manufacturing industry provides impetus to optimize their coordinated development. As the pharmaceutical manufacturing industry structure is gradually rationalized, regional trade barriers collapse, competition increases, the division of labor deepens, and market concentration increases, which will help promote the development of the entire pharmaceutical manufacturing industry, thereby narrowing the gap with the development level of the medical service industry. Eventually, the coupling coordination is improved.

From a macro perspective, macro investment, consumption, and net exports promote coupling and coordination. As the pharmaceutical manufacturing industry structure gradually advances, the increase in high value-added and high intelligence density industries will further promote the reform of the pharmaceutical supply side, increase residents' consumer demand and net exports, optimize the service level of the medical service industry, and deepen interindustry information integration to promote the coordinated development of coupling. In addition, the more reasonable the industrial structure of the pharmaceutical manufacturing industry is, the more it will attract investment from the government and foreign companies, which will help improve its R & D capabilities, deepen its connection with the medical service industry, and ultimately promote the improvement of coupling and coordination.

## Research Design

### Construction of A Model of the Coupling and Coordinated Development of the Pharmaceutical Manufacturing Industry and the Medical Service Industry

#### Construction of an Index System to Measure the Coupling Coordination Degree

Establishing a scientific and reasonable indicator system is essential to accurately measuring the degree of coordinated development between the medical service industry and the pharmaceutical manufacturing industry. Based on the basic principles of comprehensiveness, scientific methods, hierarchy, and operability, this article establishes an evaluation index system with four dimensions: industrial scale, economic benefits, social contribution, and growth potential (see Table 1). The industrial scale reflects the comprehensive performance of an industry in terms of fixed asset investment and economic aggregation, reflecting the comprehensive development level of the industry in a region; industrial benefits reflect the links between regional industries through indicators such as profit and labor productivity; and industrial contribution is the industry's contribution to the national economy. The contribution made represents the impact of industrial development on the economy and society; industrial potential represents the direction in which the industry is developing and, to a certain extent, represents the industrial development trend. The indicator data come from the EPS database, the “China Health Statistics Yearbook,” the “China Pharmaceutical Manufacturing Development Research Report,” and provincial statistical yearbooks.

**Table 1:** Index system to measure the coupling and coordination degree in the development of the pharmaceutical manufacturing industry and the medical service industry.

**Subsystem**	**First level indicator**	**Secondary indicators**	**Index explanation**	**Unit**
Pharmaceutical Manufacturing Subsystem	Industry scale	Fixed asset investment	Investment in fixed assets	Ten thousand yuan
		Total output value	Total output value	Billions
	Economic benefit	Total profit	Income and expenses	Yuan
		Labor productivity	Total output value/number of employees	Yuan/person
	Social Contribution	Employed population	Total number of employees	Millions
		Per capita output value	Total output value/number of people	Yuan
	Growth potential	Growth rate of total output value	(Total output value of the current year/Total output value of the previous year-1)*100	%
		The proportion of investment in the total investment of the whole society	(Fixed asset investment volume/National fixed asset investment volume)*100	%
Medical service industry subsystem	Industry scale	Fixed asset investment	Total investment in fixed assets	Ten thousand yuan
		Total output value	Sum of output value (total expenditure)	Billions
	Economic benefit	Total profit	Hospital income-hospital expenditure	Yuan
		Labor productivity	Total output value/number of employees	Yuan/Person
	Social Contribution	Employed population	Total number of employees	Millions
		Employed population	Government health expenditure + hospital income/ total number of people in society	Yuan
	Growth potential	The growth rate of total output value	(Total output value of the current year/Total output value of the previous year-1)*100	%
		The proportion of investment in the total investment of the whole society	(Investment in fixed assets/National investment in fixed assets)*100	%

#### Standardized Processing of Data

Standardization eliminates the influences of the indicator dimension and the quantity dimension. When there are positive and negative indicators in the system, the standardized formula is:


(1) $$\begin{array}{l} U_{\text{ijt}}=(u_{ijt}-\min u_{ijt})/(\max u_{ijt}-\min u_{ijt})\\ U_{\text{ijt}}=(\max u_{ijt}-u_{ijt})/(\max u_{ijt}-\min u_{ijt}) \end{array}$$

whereas the standardized *u*_*ijt*_ index represents the initial data, and max*u*_*ijt*_, min*u*_*ijt*_ indicates the maximum and minimum values of the jth index of province I in year t.

#### Indicator Weight

This article uses the entropy method to determine the index weight. The greater the entropy value of an indicator is, the smaller the differences in the indicator's sample value and the smaller the evaluation indicator system's overall weight, where a smaller entropy value reflects greater weight. After normalizing the data, to eliminate the influence of index logarithm minimization on the calculation results, shift *u*_*ijt*_ by a sufficiently small unit A, that is, *V*_*ijt*_ = *u*_*ijt*_ + *A* (here, take*A* = 10^−4^). To normalize the shifted data, the formula is:


(2) $${\text{p}}_{\text{ijt}}={\text{v}}_{\text{ijt}}/\sum_{\text{i-}1}^{\text{m}}{\text{v}}_{\text{ijt}}$$

The standardized matrix p_ijt_ = (p_ijt_)_m*n_ can then be obtained. To determine the entropy value *e*_*j*_ of the index*u*_*j*_, the formula is:


(3) $${\text{e}}_{\text{j}}=\text{-K}\sum_{\text{i-}1}^{\text{m}}\sum_{\text{i-}1}^{\text{T}}{\text{p}}_{\text{ijr}}{\text{lnp}}_{\begin{array}{c} \text{ijr} \end{array}}$$

where K = 1/ln(mT). Finally, determine the weight of indicator*u*_*j*_, and the formula is:


(4) $${\text{w}}_{\text{j}}={\text{(1-e}}_{\text{j}}\text{)}/\sum_{\text{j-1}}^{\text{n}}{\text{1-e}}_{\text{j}}$$

where $W_{j}\in \left[0,1\right]\text{,}\sum_{\text{j-1}}^{\text{n}}{\text{w}}_{\text{j}}\text{=1}$. After processing by the entropy method, a comprehensive index of the level of coupling and coordinated development of the medical service industry and the pharmaceutical manufacturing industry is obtained:


(5) $${\text{u}}_{\text{it}}\text{=}\sum_{\text{j-1}}^{\text{n}}{\text{w}}_{\text{j}}{\text{u}}_{\text{jit}}$$

where *u*_*ijt*_ is the standardized index, *w*_*j*_ is the weight of each indicator, and $\sum_{j-1}^{n}w_{j}=1$.

#### Coupling Coordination Degree Model

Combining related concepts in physics, the degree of coupling coordination is an index that describes the degree of influence of multiple subsystems in the production of useful mutual utility, and its expression is in Equation 6.


(6) $$C=n[(u_{1},u_{2},\ldots ,u_{n})]/\prod \left(u_{i}+u_{j}\right)$$

To quantitatively analyze the coordinated development of the coupling between the pharmaceutical manufacturing industry and the medical service industry, two systems coupling degree models based on Equation (6) are constructed as follows:


(7) $$C_{i}^{t}=2\sqrt{M_{i}^{t}▪S_{i}^{t}}/\left(M_{i}^{t}+S_{i}^{t}\right){\text{u}}_{\text{f}}$$

where $M_{i}^{t}$ and $S_{i}^{t}$ are the comprehensive index and composite index of the pharmaceutical industry in year t of province or region I. $C_{i}^{t}\in \left[0,1\right]$ The higher the value of C is, the better the combination of medical services and pharmaceutical manufacturing. When C=1, it is in the most optimal state, when C=0, it is irrelevant.

Although Equation (7) can measure the coordinated development of the medical service industry and the pharmaceutical manufacturing industry to a certain extent, it does not truly reflect the development level of them both. When the medical service industry and the pharmaceutical manufacturing industry are both at a low level of development, the degree of coupling coordination would show a false highly coupled and coordinated development state. Therefore, this paper further constructs the coupling coordination degree model (see Equation 8).


(8) $$D_{i}^{t}=\sqrt{C_{i}^{t}▪T_{\text{i}}^{\text{t}}},T_{\text{i}}^{\text{t}}=\alpha {\text{M}}_{\text{i}}^{\text{t}}+\beta S_{\text{i}}^{\text{t}}$$

$D_{\text{i}}^{\text{t}}$ is the degree of coupling coordination, and T_i_ is a composite index reflecting the overall development level of the medical service industry and pharmaceutical manufacturing industry, a=b=0.5. Based on the value of D, referring to the research of Hu et al. (26), the degree of coupling coordination is divided into different levels, as shown in Table 2.

**Table 2:** Classification of coupling coordination degree.

**Coupling coordination** **degree (D)**	**Coordination level**	**Difference value**	**Coupling coordination type**
0 ≤ *D* < 0.2	Serious disorder	u_1_ − u_2_ > 0.03	Severely maladjusted with medical service industry development lagging type IA
		u_2_ − *u*_1_ > 0.03	Severely maladjusted with pharmaceutical manufacturing industry development lagging type IB
		|u_2_ − *u*_1_| ≤ 0.03	Severely imbalanced synchronous type IC
0.2 ≤ *D* < 0.4	Barely coupled coordination	u_1_ − u_2_ > 0.03	Barely coupled with medical service industry development lagging type IIA
		u_2_ − *u*_1_ > 0.03	Barely coupled with pharmaceutical manufacturing industry development lagging type IIB
		|u_2_ − *u*_1_| ≤ 0.03	Barely coupled synchronous type IIC
0.4 ≤ *D* < 0.5	Primary coupling coordination	u_1_ − u_2_ > 0.03	Primary coupled with medical service industry development lagging type IIIA
		u_2_ − *u*_1_ > 0.03	Primary coupled with pharmaceutical manufacturing industry development lagging type IIB
		|u_2_ − *u*_1_| ≤ 0.03	Primary coupled synchronous type IIC
0.5 ≤ *D* < 0.8	Good coupling and coordination	u_1_ − u_2_ > 0.03	Well-coupled with medical service industry development lagging type IVA
		u_2_ − *u*_1_ > 0.03	Well-coupled with pharmaceutical manufacturing industry development lagging type IVB
		|u_2_ − *u*_1_| ≤ 0.03	Well-coupled synchronous type IVC
0.8 ≤ *D* ≤ 1	High-quality coupling coordination	u_2_ − *u*_1_ > 0.03	High-quality coupled with medical service industry development lagging type VA
		|u_2_ − *u*_1_| ≤ 0.03	High-quality coupling with pharmaceutical manufacturing industry type VB
		|u_2_ − *u*_1_| ≤ 0.03	High-quality coupling synchronous type VC

#### The Characteristics of the Coupled and Coordinated Development Between the Chinese Pharmaceutical Manufacturing Industry and Medical Service Industry

Table 3 shows the calculation results for the comprehensive development level of the Chinese pharmaceutical manufacturing industry (u_z_), the comprehensive development level of the medical service industry (u_f_), the degree of coupling (C), the value of u_1_ − u_2_ and the degree of coupling coordination (D) from 2004 to 2015.

**Table 3:** Annual results for the coupling and coordination degree between the pharmaceutical manufacturing industry and the medical service industry.

**Years**	**Manufacturing** **development level**	**Service industry** **development level**	**Difference** **value**	**Coupling** **degree C**	**Coupling coordination** **degree D**	**Coupling** **coordination type**
2003	0.096	0.059	0.037	0.946	0.254	Barely coupled with medical service industry development lagging type IIA
2004	0.105	0.074	0.031	0.954	0.280	Barely coupled with medical service industry development lagging type IIA
2005	0.110	0.080	0.03	0.962	0.293	Barely coupled synchronous type IIC
2006	0.112	0.086	0.026	0.996	0.297	Barely coupled synchronous type IIC
2007	0.120	0.171	−0.051	0.911	0.331	Barely coupled with pharmaceutical manufacturing industry development lagging type IIB
2008	0.129	0.139	−0.01	0.954	0.339	Barely coupled synchronous type IIC
2009	0.138	0.163	−0.025	0.954	0.356	Barely coupled synchronous type IIC
2010	0.153	0.182	−0.029	0.954	0.377	Barely coupled synchronous type IIC
2011	0.172	0.206	−0.034	0.957	0.400	Primary coupled with pharmaceutical manufacturing industry development lagging type IIB
2012	0.190	0.241	−0.051	0.966	0.428	Primary coupled with pharmaceutical manufacturing industry development lagging type IIB
2013	0.203	0.276	−0.073	0.948	0.441	Primary coupled with pharmaceutical manufacturing industry development lagging type IIB
2014	0.220	0.308	−0.088	0.943	0.459	Primary coupled with pharmaceutical manufacturing industry development lagging type IIB
2015	0.230	0.337	−0.107	0.931	0.469	Primary coupled with pharmaceutical manufacturing industry development lagging type IIB
2016	0.248	0.373	−0.125	0.930	0.487	Primary coupled with pharmaceutical manufacturing industry development lagging type IIB

Table 3 shows that the degree of coupling coordination between the pharmaceutical manufacturing industry and the medical service industry has an increasing trend, from the barely coupled medical service industry development lagging type IIA to the primary coupled pharmaceutical manufacturing industry lagging development type IIB. By comparison, *u*_1_ and *u*_2_, the development level of the Chinese pharmaceutical manufacturing industry and medical service industry, respectively, from 2003 to 2016, showed an increasing annual trend. Before 2006, the pharmaceutical manufacturing industry's development level was rising faster than that of the medical service industry, while after 2006, it lagged behind, and its growth rate was significantly lower than that of the medical service industry; in this period, the gap between the two industries was increasing annually. It can be inferred that because the development speed of the medical service industry far exceeds that of the pharmaceutical manufacturing industry, the degree of coupling and coordination between the medical service industry and the pharmaceutical manufacturing industry in Chinese remains in an unreasonable state of coordination after more than 10 years of development. The coupling degree between the Chinese pharmaceutical manufacturing industry and the medical service industry from 2003 to 2016 ranged from 0.911 to 0.966, remaining at a relatively high level. The degree of coupling and coordination between the Chinese pharmaceutical manufacturing industry and the medical service industry increased from 0.254 to 0.487 from 2003 to 2016, from the barely coupled coordination type to the primary coupling coordination type, showing improvement.

#### Development Dynamics for the Coupling and Coordination of the Chinese Pharmaceutical Manufacturing Industry and Medical Service Industry

**Figure 1:**
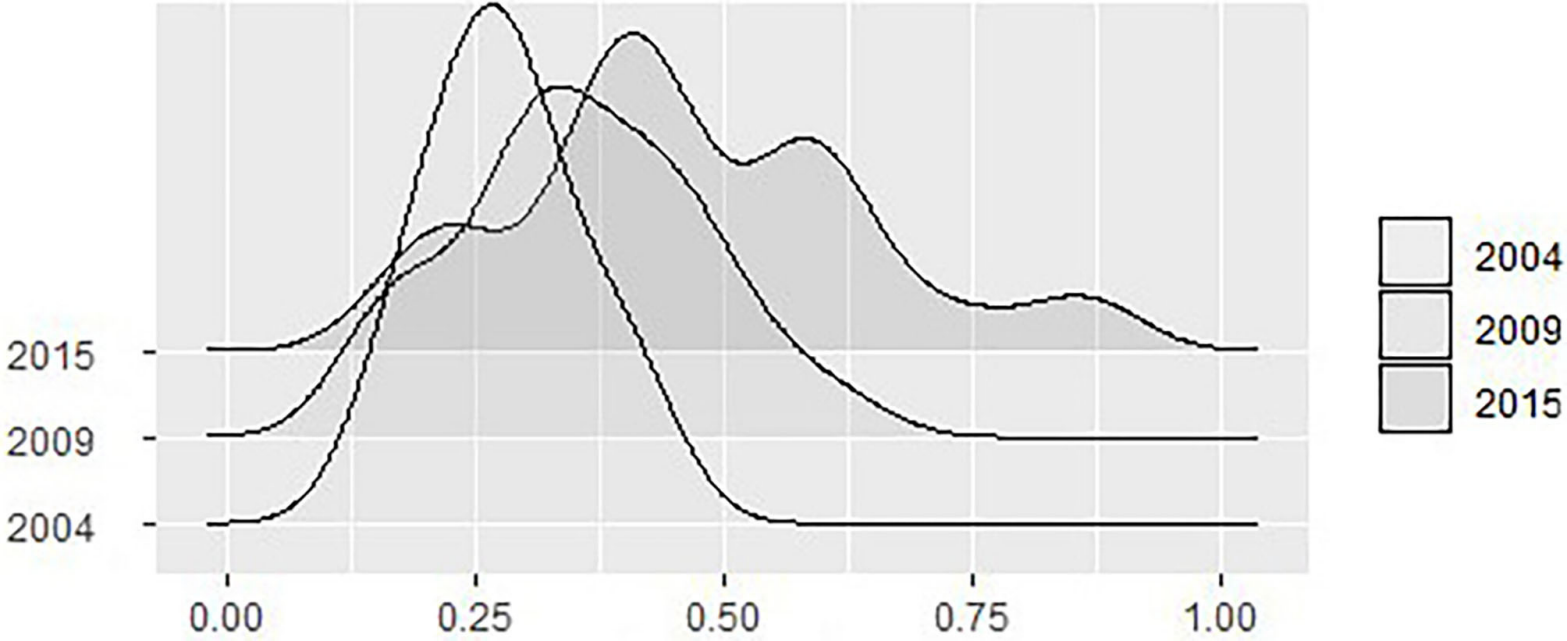
The trend in national coupling coordination degree.

We further use the kernel density function to describe the coupling coordination degree's development dynamics, as shown in Figure 1.

We selected the years 2004, 2009, and 2015 for our analysis. The graph's left and right movement in Figure 1 represents the range of variation in the degree of industrial coupling and coordination; the kurtosis describes the trend in industrial coordinated development; and the shape describes the degree of convergence and divergence in the coordinated development of the industry. It can be seen from Figure 1 that first, the overall trend in the coupling coordination degree tends to the right, indicating that the coupling coordination degree level has improved overall; second, the kurtosis of the coupling coordination degree changes from a sharp peak to a broad peak, indicating that the gap in coordinated development is gradually widening; finally, the area to the left end of the figure gradually decreases, indicating that the pharmaceutical manufacturing and medical service industries are developing more quickly. The degree of coupling and coordination is increasing annually, the two industries are developing more quickly, and thus the gap in coupling and coordination between regions is also increasing annually.

### Measurement of Upgrading in the Industrial Structure of the Pharmaceutical Manufacturing Industry

To measure industrial structure upgrading, this article refers to Qan et al. (27) and dynamically characterizes pharmaceutical manufacturing industry change considering two aspects: advanced industrial structure and industrial structure rationalization.

Based on the research of Fu et al. (28), this article adopts 6 subsectors for China's pharmaceutical manufacturing industry (excluding the veterinary drug manufacturing industry because the output value data of the veterinary manufacturing industry are missing and its proportion is small) according to production technology efficiency. Based on technological content and product value, they are divided into low-, medium-, and high-end technology industries. The manufacturing of sanitary materials and medical supplies and the manufacturing of chemical raw medicines are classified as low-end technology industries. The processing and manufacturing of Chinese patent medicines and Chinese medicine decoction are classified as mid-end technology industries. Biological medicine manufacturing and chemical preparation manufacturing are high-end technological products (28). The index of total industrial output value (the price of the year) is used to construct the advanced index for the pharmaceutical manufacturing industry structure, as shown in Equation (9):


(9) $$GJ=\frac{Y_{\text{s}}}{Y_{\text{m}}}$$

where *GJ* is the advanced index of pharmaceutical manufacturing industry structure, *Y*_*s*_ is the sum of the manufacturing output value of chemical drug preparation manufacturing and biological drug manufacturing, and *Y*_*m*_ is the total output value of the pharmaceutical manufacturing industry. The rise of *GJ* value indicates that the pharmaceutical manufacturing industry's industrial structure is tending to develop in the direction of high-efficiency pharmaceutical manufacturing.

Based on the research of Qian et al. (27), this paper adopts the degree of structural deviation as a measure of industrial structure rationalization, and the calculation formula is shown in Equation 10.


(10) $$HL=\sum_{1}^{\text{n}}\left|\frac{{\;}^{Y_{i}}\;╱\;{\;}_{L_{i}}}{{\;}^{Y}\;╱\;{\;}_{L}}-1\right|=\sum_{1}^{\text{n}}\left|\frac{{\;}^{Y_{\text{i}}}\;╱\;{\;}_{Y}}{{\;}^{L_{i}}\;╱\;{\;}_{L}}-1\right|$$

where *HL* is the rationalization of the industrial structure of the pharmaceutical manufacturing industry, *Y* and *L* are the total industrial output value and employed population, respectively, *i* is the industry, and *n* is the number of industry sectors. According to classical economic theory, when *HL* is equal to zero, the pharmaceutical manufacturing subindustries have equal productivity, and the economy is in equilibrium. When *HL* is not equal to zero, the pharmaceutical manufacturing industry deviates from the equilibrium state, and the greater the value is, the higher the deviation, and the more unreasonable the pharmaceutical manufacturing industry structure.

Finally, this article draws on the Zhang and Wang (29) to address the non-dimensional processing of the advanced index and rationalization index of the pharmaceutical manufacturing industry structure. The higher the advanced index of the pharmaceutical manufacturing industry is, the higher the advanced level of the industrial structure and the upgrading of the pharmaceutical manufacturing industry.


(11) $$GJ_{\text{it}}=\frac{GJ_{it}-\min GJ_{it}}{\max GJ_{it}-\min GJ_{it}}$$

(12) $$HL_{\text{it}}=\frac{HL_{it}-\min HL_{it}}{\max HL_{it}-\min HL_{it}}$$

## Model Construction and Variable Selection

### Model Building

Based on the above analysis, we build a measurement model:


(13) $$Y_{\text{it}}=\beta_{0}\text{+}\beta_{\text{1}}GJ_{\text{it}}+\beta_{2}HL_{\text{it}}+\sum_{j=\text{3}}^{5}\beta_{j}Control_{j,it}+\varepsilon_{it}$$

where *Y*_it_ is the coupling degree between the pharmaceutical manufacturing and medical service industry, *GJ*_it_ is the advanced index of the pharmaceutical manufacturing industry structure *HL*_it_ is the industrial structure rationalization index of the pharmaceutical manufacturing industry. Control, β, *i* and *t* are the regression coefficients, the name of the province, the years, and the random error term, respectively. To explore the role of the pharmaceutical manufacturing industry's industrial structure upgrade on different stages in the evolution of the coupling coordination degree and the influence of the general conditional distribution law, this paper adopts the quantile regression method. It uses the weighted average of the residual's absolute value as the minimization objective, and the entire condition is distributed. According to the medical service industry's different development levels, we explore the impact of industrial structure advancement and the rationalization index on the degree of coupling coordination.

#### Quantile Regression Model

**Figure 2:**
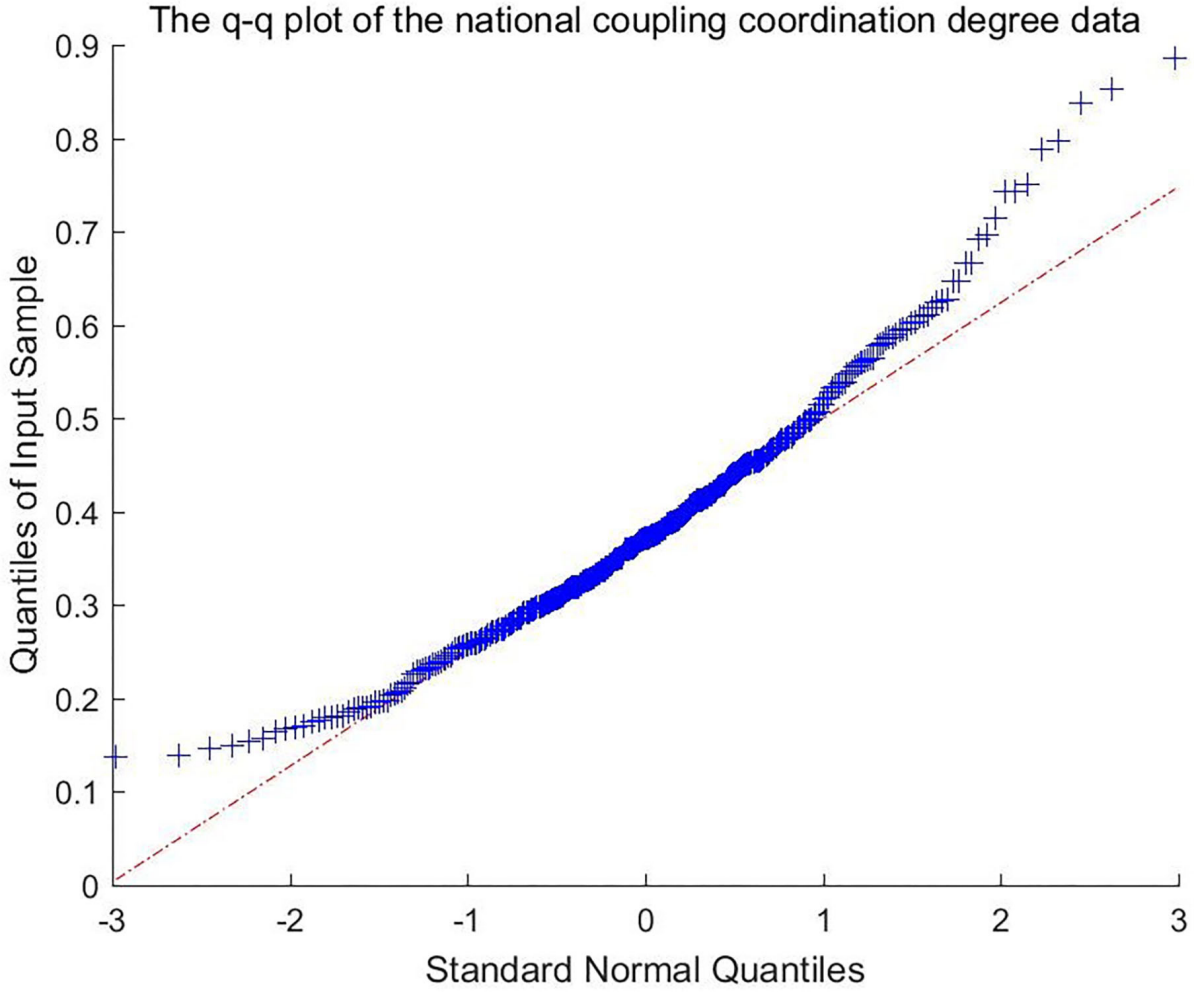
The q-q plot of the national coupling coordination degree data.

According to the previous analysis, upgrading the industrial structure of the pharmaceutical manufacturing industry can drive its development, reduce its development gap with the medical service industry, and promote their coordinated development. In this essay, the main purpose is to identify the affects brought by the development of pharmaceutical manufacturing industry to coordinated development. However, the upgrading of the industry will not uniformly affect the coordinate development. Before any regression methods are applied the data features should be observed first. The following Q-Q plot and table demonstrates the data feature (as shown in Figure 2). The data illustrates obviously fat-tail feature, thus it is rational to apply the quantile regression rather than traditional linear regression.

The quantile of 0.05, 0.1, 0.25, 0.5, 0.75, 0.9, 0.95 will be selected to test the correlation between pharmaceutical manufacturing industrial development and coordinated development promotion. More than one quantile were selected to observe the correlation between them under different testing level. It is expected to show similar results under all these altered criterion.

The quantile regression model is:


(14) $$\text{quan}t_{\tau }(Y_{it}|x_{it})=x_{it}\beta_{\tau }$$

where τ is the quantile (0 <τ <1), β_τ_ is the quantile regression coefficient, which can be obtained by LAD (minimize absolute deviation), *x* and β_τ_ is vector of *K* * 1, (*i* = 1, 2, …, *n, t* = 1, 2, …, *T*). The function of LAD can be expressed as


(15) $$\begin{array}{l} \beta_{\tau }=\arg\min\{\sum_{i=1:Y_{\text{it}}>x_{it}\beta_{\tau }}^{n}\left|Y_{\text{it}}\text{-}x_{it}\right|\\ \;+\sum_{i=1:Y_{\text{it}}\leq x_{it}\beta }^{n}\left|Y_{\text{it}}\text{-}x_{it}\right|\} \end{array}$$

#### Threshold Regression Model

To further explore the non-linear development relationship between industrial structure advancement and coupling coordination degree according to the panel threshold model proposed by Hansen, this paper constructs a panel threshold regression model of industrial structure advancement and coupling coordination degree, which reads as


(16) $$\begin{array}{l} {\text{Y}}_{\text{it}}=\theta_{\text{1}}HL_{\text{it}}\cdot \text{I}(\text{Fwy}\leq \text{r})+\theta_{\text{2}}HL_{\text{it}}\cdot \text{I}(\text{Fwy}>\text{r})+\\ \;\beta_{\text{1}}GJ_{\text{it}}+\beta_{2}{\text{JJ}}_{it}+\beta_{3}ZB_{it}+\beta_{4}ZF_{it}+\beta_{\text{0}}+\varepsilon_{\text{it}} \end{array}$$

where JJ_*it*_ is the synergy agglomeration index of the pharmaceutical manufacturing industry and the medical service industry, *ZB*_*it*_ is provincial capital stock, *ZF*_*it*_ is controlled by the government, *Fwy* is the threshold variable of the medical service industry level. This article uses the number of beds in medical and health institutions per thousand population to represent, r is the unknown threshold, I(▪) is the indicator function, which satisfies the conditions in brackets, I = 1, otherwise, I = 0. θ_1_ and θ_2_ are the estimated values of the influence coefficient of the advanced pharmaceutical manufacturing industry structure on the coupling coordination degree before and after the threshold value is reached, β_0_, β_1_, β_2_, β_3_, β_4_ are other coefficients to be estimated, and ε_it_ is the random disturbance term.

### Variable Selection

Coupling coordination degree between the pharmaceutical manufacturing industry and the medical service industry (Y): Calculated based on the pharmaceutical manufacturing industry's development coupling coordination index system and the medical service industry. All data come from the EPS database, the “China Health Statistics Yearbook,” “The Development of China's Pharmaceutical Manufacturing Industry Research Report” and provincial statistical year books.

Core explanatory variables: Pharmaceutical manufacturing industry structure advanced index (GJ) and rationalization index (HL). All data comes from “China Industrial Enterprise Database,” using Nie Huihua's method, in the 2004–2015 industrial enterprise database, excluding firms with outliers for sales and the number of employees, missing total assets, and having fewer than 8 employees. Missing data were calculated using the moving average method to fill in the missing values.

Control variables:

(1) Synergistic agglomeration of the pharmaceutical manufacturing industry and the medical service industry: Drawn the research of Zhang et al. (18), uses location entropy to measure the agglomeration index of the medical service industry and the pharmaceutical manufacturing industry (see Equation 17).
  (17) $$JJ=(1-\frac{\left|JJ_{2}-JJ_{1}\right|}{JJ_{1}+JJ_{2}})$$
  where *JJ* is the cooperative agglomeration index, *JJ*_1_ is the location entropy of the pharmaceutical manufacturing industry's output value, and *JJ*_2_ is the location entropy of the output value of the medical service industry. Based on the theories of the value chain, transaction cost, and social network, the collaborative agglomeration of the pharmaceutical manufacturing industry and the medical service industry accelerates the flow of production factors, forms economies of scale, accelerates information communication, reduces transaction and production costs, and promotes coordinated and coupled development.
(2) Provincial capital stock (ZB): Based on the research of Zhang et al. (18), expressed as the logarithm of the proportion of fixed assets in GDP.
(3) Government control (ZF): Based on national (2019) research, expressed as the proportion of government fiscal expenditure in GDP. The control variable data are all from the “China Statistical Yearbook,” “China Health Statistics Yearbook,” etc. See Table 4 for analysis.

**Table 4:** Statistical description of main variables.

**Variable**	**Observations**	**Average** **value**	**Standard** **error**	**Minimum**	**Max**
y	348	0.386	0.136	0.137	0.885
GJ	348	0.490	0.217	0	1
HL	348	0.080	0.084	0	1
ZB	348	0.638	0.212	0.253	1.328
JJ	348	0.776	0.188	0.109	1.000
ZF	348	0.203	0.091	0.079	0.627
Fwy	348	3.812	1.274	1.500	7.550

This paper takes the panel data of 29 provinces in China from 2004 to 2015 (given the severe lack of data in the Tibet Autonomous Region and Hainan Province, they are not included) as a sample and uses R3.6 software to perform fixed effects, quantile, and Bayes quantiles number regression analysis.

### Empirical Results and Analysis

In order to accurate the regression results, before the correlation analysis, the panel unit root test is performed on the model variables to judge each variable's stability. This paper uses the Fisher-ADF test and finds that there is no unit root for each variable; that is, all variables are stable. This paper further tests the related variables' multicollinearity and found that the VIF (variance inflation factor value) is between 1.030 and 1.733 (see Table 5), which is much <10, indicating that multicollinearity between variables is in the controllable range.

**Table 5:** VIF (variance inflation factor) value of main variables.

**Variable**	**GJ**	**HL**	**ZB**	**ZF**	**JJ**
VIF value	1.300	1.040	1.733	1.642	1.030

### Quantile Regression Model

**Figure 3:**
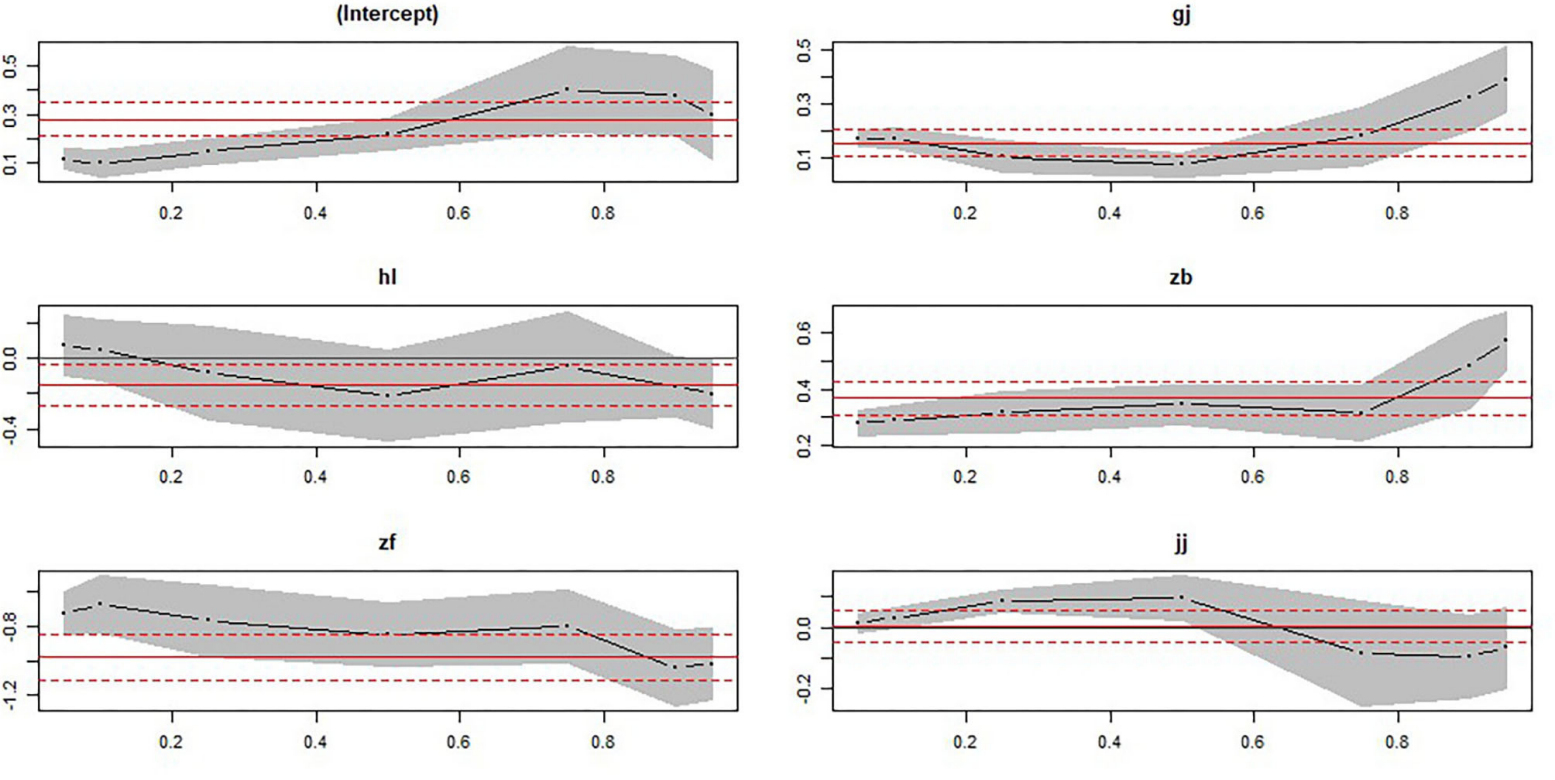
Correlation coefficient change graph.

To verify the results of the traditional panel fixed effects and make effective comparisons with the quantile model, this paper uses the fixed effects model as the reference result of the quantile regression model. Seven representative quantiles of 0.05, 0.1, 0.25, 0.5, 0.75, 0.9, and 0.95 were selected, and FE (fixed effects model) was added for comparison. The correlation analysis results are shown in Table 6, and the correlation coefficient changes are shown in Figure 3. The wald test is also applied after the regression. The F-value is 4.29 and *p*-value is 0.0003, which supported the rationality of this regression.

**Table 6:** Test results for quantiles.

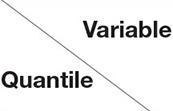	**GJ**	**HL**	**ZB**	**ZF**	**JJ**	**Constant** **term**
FE	0.097^**^	−0.057	0.304^***^	0.128	−0.100^***^	0.190^***^
	(3.080)	(−1.450)	(10.16)	(1.580)	(−2.870)	(5.500)
0.05	0.173^***^	0.075	0.279^***^	−0.724^***^	0.015	0.116^***^
	(9.209)	(0.731)	(10.8287)	(−9.711)	(−0.788)	(4.539)
0.1	0.174^***^	0.043	0.289^***^	−0.670^***^	0.031	0.100^***^
	(7.757)	(0.419)	(9.738)	(−6.916)	(1.579)	(3.135)
0.25	0.107^**^	−0.081	0.316^***^	−0.765^***^	0.086^***^	0.147^***^
	(2.898)	(−0.537)	(7.144)	(−6.160)	(4.126)	(4.653)
0.5	0.078^**^	−0.212	0.345^***^	−0.846^***^	0.096^**^	0.218^***^
	2.858	(−1.447)	(8.236)	(−8.146)	(2.179)	(5.532)
0.75	0.183^***^	−0.048	0.314^***^	−0.798^***^	−0.085	0.400^***^
	(2.918)	(−0.264)	(5.207)	(−6.543)	(−0.779)	(3.534)
0.9	0.328^***^	−0.160	0.484^***^	−1.038^***^	−0.096	0.377^***^
	(4.262)	(−1.330)	(5.089)	(−7.643)	(−1.130)	(3.598)
0.95	0.392^***^	−0.202^**^	0.572^**^	−1.017^***^	−0.066	0.297^***^
	(5.061)	(−2.047)	(8.604)	(−7.774)	(−0.835)	(2.731)

** and *** *indicate significance at the 10% and 5% levels. The values in the brackets are the values obtained by bootstrapping. The results are obtained by R software. The results below are the same*.

Table 6 shows that, in general, the fixed effects model and the impact coefficients of the pharmaceutical manufacturing industry structure advanced index at each quantile are positive and pass the 1% significance level test, which means that since 2004, the industrial structure advanced index of the pharmaceutical manufacturing industry has had a positive impact on the degree of coupling and coordination. By observing the changing trend in the index coefficients of the pharmaceutical manufacturing industry structure at each quantile, it is found that the coefficients of the pharmaceutical manufacturing industry structure advancement index generally show an increasing trend, and the coupling coordination degree increases from 7.8 to 39.2%. When other conditions are controlled, at different quantile levels of the coupling coordination degree, the pharmaceutical manufacturing industry's advanced structure and coupling coordination degree are positively correlated. From the previous analysis, it can be seen that the development of the pharmaceutical manufacturing industry lags behind that of the medical service industry, and this is the main restriction on the degree of coupling and coordination. The advanced structure of the pharmaceutical manufacturing industry reflects an optimized output and resource utilization structure, optimized resource allocation, and improved production efficiency and improved economic output, significantly promoting its efficient development and thereby narrowing the gap its development level and that of the medical service industry, ultimately promoting coupling and coordination between the two industries. Specifically, when the degree of coupling and coordination is low, the pharmaceutical manufacturing industry and the medical service industry are both at a low level of development, and there is little between the two. With improvement of the pharmaceutical manufacturing industry toward an advanced level, the gap between the development levels of the two industries gradually increases, weakening the effect of advanced pharmaceutical manufacturing on the degree of coupling and coordination. When the degree of coupling and coordination is high, the gap between the two industries is also high. Upgrading the pharmaceutical manufacturing industry structure can significantly reduce the gap between the two industries' development levels and thus have a significant effect on promoting the degree of coupling and coordination. The coefficient of 0.097 in the fixed effects model is positive, which further proves that the pharmaceutical manufacturing industry's advanced structure can promote its collaborative and coordinated development with the medical service industry.

Table 6 also shows that the coefficient of the pharmaceutical manufacturing industry structure rationalization index decreased from 0.075 in the low quintile to −0.202 in the high quintile and only reached significance in the high quintile of 0.95, indicating that the level of structural rationalization in the pharmaceutical manufacturing industry has a significant inhibitory effect on cities where the degree of coupling coordination is on the verge of imbalance but no significant effect on cities with low coupling coordination. This conclusion shows that the level of rationalization in the pharmaceutical manufacturing industry structure plays a vital role in developing the Chinese health industry. Rationalizing the pharmaceutical manufacturing industry structure would represent an essential breakthrough in developing urban health industries that are currently unbalanced and underdeveloped.

Among other control variables, the correlation coefficients of fixed asset investment variable all pass the 5% significance test, and all quantile levels are positive, indicating that fixed asset investment can effectively promote the pharmaceutical manufacturing and medical service industries. The collaborative development of coupling and the coordinated development of China's economy is consistent with China's current investment promotion of the real economy. Capital factors play a more significant role in promoting highly-coupled and coordinated areas, consistent with the Chinese policy of vigorously supporting industrial cluster development. The correlation coefficient of the collaborative agglomeration level of the pharmaceutical manufacturing industry and the medical service industry on their coupling and coordination degree shows a non-linear state and only reaches significance at the 25 and 50% quantiles, and the correlation coefficient is from a low quantile. The positive influence of the high quantile point turns into a negative influence, which also confirms the related theory of industrial agglomeration: in the early stage of industrial development, collaborative agglomeration helps the coupling and coordinated development of industries, but as the level of agglomeration increases, the crowding effect produced by excessive agglomeration will inhibit the coordinated development of coupling between industries. The coefficient of government control for this variable is negative at each quantile level and passes the 1% significance test, which shows that since 2004, government fiscal expenditure has not effectively promoted the coordinated development of the pharmaceutical manufacturing industry and the medical service industry.

### Threshold Effect Test and Threshold Value Determination

The above empirical results show that the pharmaceutical manufacturing industry structure advancement index may have a non-linear effect on the degree of coupling and coordination, so this article further uses a threshold model to verify its non-linear effect. This paper estimates single- and double-threshold models to find the minimized model's squared residual and the threshold value. The bootstrap method is used to simulate the asymptotic distribution of the F statistic at the critical value, and the number of bootstraps is 1,000. To explore the optimal threshold number, the single threshold, double threshold, and triple threshold were estimated separately, and the *P*-value and F value obtained by the “self-sampling method” and the critical value for each significance level are shown in Table 7.

**Table 7:** Test results for the existence of the coupling coordination degree threshold for advanced pharmaceutical manufacturing.

**Threshold variable**	**Critical value**				
	***F*-value**	***P*-value**	**10%**	**5%**	**1%**
Single threshold	107.07^***^	0.00^***^	28.79	35.78	52.01
Double threshold	53.89^***^	0.00^***^	21.11	25.22	35.64
Triple threshold	17.00	0.69	50.24	59.63	87.38

*The P-value and critical value are obtained by repeated sampling, with bootstrap = 1,000;*

*** *means significant at the 1% level*.

Table 7 shows that the *P*-values of the single and double threshold both pass the 1% significance test, and the effect is significant. In contrast, the triple threshold is not significant, indicating that the advanced index of the pharmaceutical manufacturing industry structure has a dual-threshold effect on the degree of coupling coordination. Double threshold model for specific analysis.

**Figure 4:**
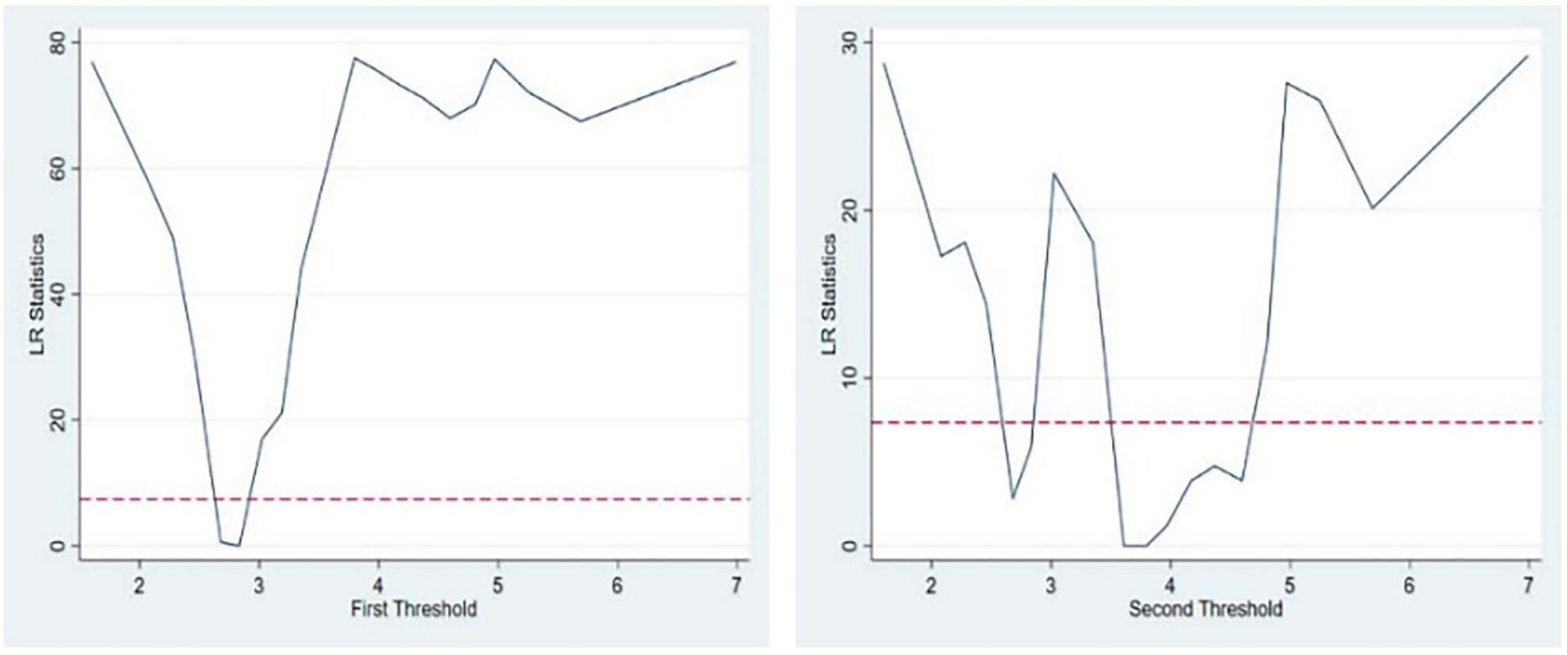
Threshold value diagram.

The two thresholds and the corresponding 95% confidence intervals are shown in Table 8. It can be seen from Table 8 that the two thresholds divide the development level of the medical service industry into three intervals, (BMI3.190), (3.190BMI3.800), (3.80BMI). With the help of the relief ratio function diagram of the threshold model regression, it is easy to understand the process for estimating the threshold value and constructing the confidence interval. The likelihood ratio function is shown in Figure 4.

**Table 8:** Threshold.

	**Estimated value**	**95% confidence interval**
Threshold r1	3.190	[3.025,3.479]
Threshold r2	3.800	[3.479,3.970]

The estimated results for the panel threshold model are shown in Table 8. From the threshold regression results, it can be seen that the impact of the advanced medical manufacturing industry structure on the degree of coupling and coordination has different effects at different medical service industry development levels. When the development of the medical service industry is at a low level, the pharmaceutical manufacturing industry structure advanced index has a negative effect on the degree of coupling and coordination, and the estimated coefficient is−0.10; when the development of the medical service industry is at a medium level, the index has no significant effect on the degree of coupling coordination; when the development of the medical service industry is at a high level, the index has a positive effect on the degree of coupling coordination, and the coefficient is estimated to be 0.15 (Table 9). An increase in the coefficient has a significant effect on coupling coordination. The threshold effect results fully prove that a higher-level pharmaceutical manufacturing industry structure has a threshold effect on its coupling and coordination with the medical service industry. According to 2018 statistics, the service industry's development level in 31 provinces across the country exceeded 5.24. China is clearly in the development stage, where improving the structure of the pharmaceutical manufacturing industry can significantly improve the degree of coupling and coordination.

**Table 9:** Threshold correlation coefficient.

**Variable**	**Coefficient** **estimate**
Pharmaceutical manufacturing rationalization index	−0.07^**^
	(−2.06)
Provincial capital stock	0.16^***^
	(6.09)
Government control	0.09
	(0.93)
Collaborative gathering	−0.08^**^
	(−3.01)
Advanced pharmaceutical manufacturing 1	−0.10^***^
	(−3.18)
Advanced pharmaceutical manufacturing 2	0.04
	(1.46)
Advanced pharmaceutical manufacturing 3	0.15^***^
	(2.73)
R	0.91
Number of samples	348

***, ** *represent significance at the levels of 0.05 and 0.1. The t value of each variable is in parentheses*.

## Robustness Analysis

To ensure the accuracy of its conclusions, this paper adopts the Bayesian adaptive lasso quantile method to re-estimate the main regression results and conduct a robustness test.

**Figure 5:**
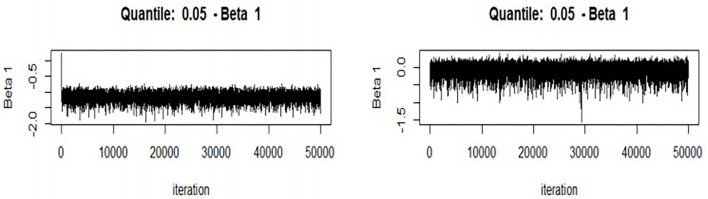
Markov chain fitting degree diagram.

For numerical stability, we standardized the prediction function and the dependent variable and include some assumptions. For example, the default quantile is “median,” and the default form is “adaptive lasso.” Finally, the number of MCMC iterations must be set. The check after acceptance will show whether 50,000 is sufficient to find convergence in the MCMC; if not, the value must be increased (Figure 5). Figure 3 shows the trace graph used to check MCMC convergence visually. As shown in Figure 3, the MCMC sampler quickly moves to a smooth distribution and mixes evenly, which indicates that it passes the check on the convergence of the MCMC (for reasons of space, only the above two figures are listed), indicating that the results are robust.

The Bayesian adaptive lasso quantile model (BALQR) is based on independent and uniform error assumptions, allowing different penalty parameters to be used for different regression coefficients to expand the Bayesian lasso quantile regression Gibbs sampling and simulate the parameters from the posterior distribution (Table 10).

**Table 10:** Bayesian quantile regression results (ndraw = 50,000).

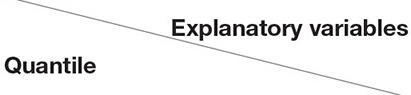		**GJ**	**HL**	**ZB**	**ZF**	**JJ**	**Constant term**
0.050	Estimate	0.198	−0.057	0.296	−0.425	0.061	−1.159
	lower	−0.059	−0.465	−0.065	−0.838	−0.101	−1.459
	upper	0.466	0.167	0.678	−0.073	0.307	−0.925
0.100	Estimate	0.223	−0.049	0.392	−0.460	0.064	−0.945
	lower	0.010	−0.351	0.095	−0.774	−0.068	−1.139
	upper	0.424	0.132	0.661	−0.172	0.239	−0.774
0.250	Estimate	0.149	−0.079	0.472	−0.508	0.105	−0.589
	lower	−0.012	−0.320	0.268	−0.765	−0.020	−0.745
	upper	0.324	0.082	0.683	−0.282	0.237	−0.436
0.500	Estimate	0.119	−0.094	0.513	−0.572	0.102	−0.134
	lower	−0.015	−0.294	0.319	−0.769	−0.045	−0.275
	upper	0.266	0.054	0.705	−0.381	0.241	0.004
0.750	Estimate	0.238	−0.072	0.476	−0.561	−0.062	0.498
	lower	−0.009	−0.265	0.233	−0.778	−0.297	0.298
	upper	0.472	0.072	0.720	−0.370	0.160	0.705
0.900	Estimate	0.393	−0.040	0.608	−0.635	−0.164	1.200
	lower	0.015	−0.215	0.199	−0.880	−0.489	0.937
	upper	0.753	0.178	0.999	−0.363	0.082	1.471
0.950	Estimate	0.449	−0.009	0.617	−0.597	−0.209	1.589
	lower	−0.040	−0.217	0.061	−0.921	−0.653	1.274
	upper	0.921	0.332	1.120	−0.141	0.101	1.973

The Bayesian quantile estimation results show that the pharmaceutical manufacturing industry structure's influence coefficient for the coupling coordination degree between it and the medical service industry remains positive. As the coupling coordination degree increases, its effect will also increase. This further verifies the conclusion that the pharmaceutical manufacturing industry's advanced industrial structure can positively promote coordinated development between the pharmaceutical manufacturing industry and the medical service industry. In addition, the industrial structure rationalization index of the pharmaceutical manufacturing industry has a negative effect on the degree of coupling and coordination, which is also consistent with the previous conclusions (due to length limitations, these are not repeated here).

## Conclusions and Policy Recommendations

This paper conducts quantile regression and threshold regression analyses on the panel data of 29 provinces and cities from 2004 to 2015 to test the impact of industrial structure upgrading in the Chinese pharmaceutical manufacturing industry on the degree of coupling and coordination and draws the following conclusions:

(1) The upgrading of the pharmaceutical manufacturing industry structure is a double-edged sword for the improvement of coupling coordination. First, the advanced structure of the pharmaceutical manufacturing industry can promote coupling coordination, but the rationalization of the structure will inhibit its development under highly coupled conditions. Second, the advancement of the pharmaceutical manufacturing industry structure has a non-linear relationship with the coordinated development of coupling. Further analysis of the threshold effect shows that the advancement in the pharmaceutical manufacturing industry structure presents a significant threshold feature for improving the degree of coupling and coordination. The specific performance is as follows: improvement in the medical service industry's development level will first restrain and then strengthen the degree of coupling coordination.
(2) Interprovincial capital stock promotes the coordinated development of coupling, and the degree to which collaborative agglomeration inhibits coordinated development is the same as that found in the quantile regression. The inhibitory effect of government control and rationalization on the degree of coupling and coordination is not significant in the threshold model. Coupling and coordination between the pharmaceutical manufacturing industry and the medical service industry is a dynamic coordination state. In the embryonic period, the pharmaceutical manufacturing industry is in an active position, its advanced structure drives the development of the medical service industry, and the industrial structure of the pharmaceutical manufacturing industry gradually changes from reasonable to unreasonable, inhibiting the coordinated development of coupling. During the stable period of interaction between the two, the medical service industry is in an active position and able to advance the pharmaceutical manufacturing industry's technological advancement; the structure changes from unreasonable to reasonable, and the inhibitory effect weakens. The difference in coupling and coordination effects is related to the level of the medical service industry: only when the medical service industry develops to a certain level can the advanced medical manufacturing industry match the medical service industry to produce a good interaction effect and promote a shift in the health industry from disorder to order. With the improvement of coupling and coordination, the influence coefficient of industrial structure rationalization presents a U-shaped change.

Therefore, the following policy recommendations are proposed. First, the government should actively guide the mergers and acquisitions and reorganization of Chinese pharmaceutical companies, optimizing the industrial layout so that prominent companies can join forces, obtain advantages of scale, and promote industry development to achieve advanced development. Second, the government should strengthen its support for scientific and technological innovation in the pharmaceutical manufacturing industry, eliminate outdated production capacity, adjust the industry structure, and improve the rationalization level of the pharmaceutical manufacturing industry to promote the development of this health industry subindustry and prevent the pharmaceutical manufacturing industry from changing its structure to have a reasonable restraining effect on the industry. Cities where the health industry's development is lagging should consider introducing high-end pharmaceutical manufacturing technology to promote its transformation and development. Third, when accelerating the implementation of the “Healthy China 2030” plan, the government should increase financial support for R&D and technological innovation, improve relevant industry regulations, transform the pharmaceutical manufacturing industry, and highlight enhancements to the supply of health products and services. The coordinated and coupled development of the pharmaceutical manufacturing industry and medical service industry should be deepened to provide strong support for the implementation of the Healthy China strategy. In this research, it is proved that quantile regression is able to represent and supervise the correlation between the development level of pharmaceutical manufacturing industry and promotion of coupling coordination. In the future researches of this topic, the optimized quantile regression such as, spatial quantile regression may provide huge help in identifying the accurate relationship between them.

It is also interesting to find that the Chinese pharmaceutical manufacturing upgrading and the coupling and coordinated development represent a non-linear relationship. Furthermore, in the realistic world the pharmaceutical manufacturing, medical services and other related industries that may affect coupling and coordinated development are very sensitive to many factors such as, technology, education, and government policies. This result in the complexity of the data, and the data feature might be changed, which requires adjustments of models. Finally, in this study, the requirement of calculation is focus on the accuracy rather than speed. The length of data is <400, and it is low frequency discrete data. Based on this situation, optimized quantile regression such as, the panel quantile regression applied by Bui (30), optimized Bayesian quantile regression applied by Myers et al. (31) and adjusted censored quantile regression applied by Wang and Chen (32).

With all these modern models this study may provide more contributions to the health industry and finally improve the quality of living experiences of citizens.

## Data Availability Statement

The original contributions presented in the study are included in the article/supplementary material, further inquiries can be directed to the corresponding author/s.

## Author Contributions

MH, CT, and HZ contributed to conception and design of the study. CT organized the database. HZ performed the statistical analysis. MH wrote the first draft of the manuscript. CT and HZ wrote sections of the manuscript. All authors contributed to manuscript revision, read, and approved the submitted version.

## Conflict of Interest

The authors declare that the research was conducted in the absence of any commercial or financial relationships that could be construed as a potential conflict of interest.

## Publisher's Note

All claims expressed in this article are solely those of the authors and do not necessarily represent those of their affiliated organizations, or those of the publisher, the editors and the reviewers. Any product that may be evaluated in this article, or claim that may be made by its manufacturer, is not guaranteed or endorsed by the publisher.

## References

[1] Wang M Gilmour S Tao C. Does scale and efficiency of government health expenditure promote development of the health industry? Int J Environ Res Public Health. (2020) 17:5529. 10.3390/ijerph17155529 32751769 PMC7432442

[2] Zhang YL Cui RM Guo GZ. Industrial policy, trade policy and industrial upgrade. Int Trade Issues. (2020) 111–28. 10.13510/j.cnki.jit.2020.07.008

[3] Wu B. One belt one road regional value chain construction and china's industrial transformation and upgrading research. Inquiry Into Econ Issues. (2020) 102–9. Available online at: http://www.cqvip.com/qk/95595x/202007/7102404146.html

[4] Tian K Dietzenbacher E Jong-A-Pin R. Measuring industrial upgrading: applying factor analysis in a global value chain framework. Econ Syst Res. (2019) 31:642–64. 10.1080/09535314.2019.1610728

[5] Fu LH. An empirical study on the relationship between my country's industrial structure advancement and economic growth. Stat Res. (2010) 27:79–81. 10.19343/j.cnki.11-1302/c.2010.08.011

[6] Deng C Cao ZW. Financial structure marketization, technical innovation and industrial structure upgrading. J Xi'an Jiaotong Univ (Soc Sci). (2020) 40:20–9. 10.15896/j.xjtuskxb.202005003

[7] Lin L Zhong X. Exploration of China's industrial structure upgrade from the perspective of ecological civilization. Ecol Econ. (2020) 36:221–5.

[8] Song L Zhang Y. Industrial transformation and upgrading of manufacturing industry driven by innovation. J Xi'an Jiaotong Univ (Soc Sci Edn). (2020) 36:37–47.

[9] Ji LY. The impact of technological innovation on the upgrading of china's manufacturing industry structure—based on the moderating effect of financing constraints. Technoeconomics. (2018) 37:30–6.

[10] Sun C. Analysis of the spatial effect of industrial structure upgrading from the perspective of industrial symbiosis. Macroecon Res. (2017) 40:114–27. 10.15896/j.xjtuskxb.202001006

[11] Jiang DC Wang CY. Foreign direct investment and the upgrading of china's manufacturing industry. Nankai J. Philos Soc Sci Edn. (2020) 32–43.

[12] Fu T Wang FF Xue YJ. Research on the matching effect of human capital and manufacturing upgrade. Stat Decis. (2020) 36:71–4. 10.13546/j.cnki.tjyjc.2020.06.015

[13] Shan QQ. The impact of collaborative innovation on the upgrading of equipment manufacturing industry structure from the perspective of spatial correlation. Syst Eng. (2020) 38:17–26. Available online at: http://www.cqvip.com/qk/93285x/202003/7102012635.html

[14] Zhou N. Research on the Coordinated Development of Manufacturing Industry and Service Industry in China (Ph.D. dissertation). Zhongnan University of Economics and Laws, Wuhan (2019).

[15] Wan ZY Ge P Zhang XL. Research on equipment manufacturing industry upgrade under the background of intelligent manufacturing. World Sci Technol Res Dev. (2018) 40:316–27.

[16] Zhang ZY. Research on improving the competitiveness of my country's advanced equipment manufacturing industry under the background of supply-side reform. Contemp Econ Manage. (2016) 38:52–56.

[17] Cai F Wang DW Qu Y. Analysis of China's industrial upgrading by the great power geese array model. Econ Res. (2009) 44:4–14.

[18] Zhang H Han AH Yang QL. Analysis of the spatial effect of the collaborative agglomeration of china's manufacturing and producer services. Quant Tech Econ Res. (2017) 34:3–20.

[19] Jiang MQ Xi QM. Industrial relevance and cooperative agglomeration between producer services and manufacturing. Nankai J Philos Soc Sci Edn. (2014) 1:153–60.

[20] Francois J Woerz J. Producer services, manufacturing linkages, and trade. Soc Sci Electron Pub. (2008) 8:199–229. 10.1007/s10842-008-0043-0

[21] Francois J Hoekman B. Services trade and policy. J Econ Lit. (2010) 8:642–92. 10.1257/jel.48.3.642

[22] Li D Lu Y Wu M. Industrial agglomeration and firm size: evidence from China. Reg Sci Urban Econ. (2012) 42:135–43. 10.1016/j.regsciurbeco.2011.07.003

[23] Lv Z Qiao L. Analysis of healthcare big data. Future Generation Computer Systems. (2020) 109:103–10. 10.1016/j.future.2020.03.039

[24] Dong BY Xu YZ Fan XM. How to achieve a win-win situation between economic growth and carbon emission reduction: empirical evidence from the perspective of industrial structure upgrading. Environ Sci Pollut Res. (2020) 27:43829–44. 10.1007/s11356-020-09883-x 32740847

[25] Lv Z Song H. Mobile internet of things under data physical fusion technology. IEEE Internet Things J. (2020) 7:4616–24. 10.1109/JIOT.2019.2954588

[26] Hu YR Tian CZ Lu P. The mechanism and empirical research on the spatio-temporal coupling effect of industrial transformation and upgrading and new urbanization. Indust Technol Econ. (2020) 39:80–7.

[27] Qan CH Zheng RG Yu DF. The impact of china's industrial structure changes on economic growth and volatility. Econ Res. (2011) 46:4–16.

[28] Fu YH Ye XS Wang ZX. The structural changes of manufacturing industry and the improvement of economic growth efficiency. Econ Res. (2016) 51:86–100. 28000739

[29] Zhang HX Wang Y. Economic system changes, industrial structure evolution and china's high-quality economic development. Econ Syst Reform. (2020) 31–37.

[30] Bui Q Wang ZH Zhang B Le HP Vu KD. Revisiting the biomass energy-economic growth linkage of brics countries: a panel quantile regression with fixed effects approach. J Clean Prod. (2021) 316:128382. 10.1016/j.jclepro.2021.128382

[31] Myers TD Negra Y Sammoud S Chaabene H Nevill AM. Discerning excellence from mediocrity in swimming: new insights using bayesian quantile regression. Eur J Sport Sci. (2021) 21:1083–91. 10.1080/17461391.2020.1808080 32772638

[32] Wang Q Chen SN. Moment estimation for censored quantile regression. Econ Rev. (2021) 40:815–29. 10.1080/07474938.2021.1889207

